# Educational needs of family physicians in the domains of health and conformity with continuing education in Fasa University of Medical Sciences

**Published:** 2015-04

**Authors:** NAHID ZARIF SANAIEY, SAHAR KARAMNEJAD, RITA REZAEE

**Affiliations:** 1 Distance Educational Planning, Center of Excellence for e-Learning in Medical Sciences, Shiraz University of Medical Sciences, Shiraz, Iran;; 2 Fasa University of Medical Science, Fasa, Iran;; 3 Quality Improvement in Clinical Education Research Center, Shiraz University of Medical Sciences, Shiraz, Iran;

**Keywords:** Needs assessment, Physicians, Family, Health, Education

## Abstract

**Introduction:**

Assessment and prioritization are the first steps of planning. According to the family physician's idea, evaluating programs in order to improve them is one of the necessities of promoting quality and increases the efficiency and effectiveness of continuing education. This study aimed to determine family physicians’ educational needs regarding health and its applicability in continuous medical education in Fasa University of Medical Sciences.

**Methods:**

In this cross-sectional study, viewpoints of 45 general physicians working at Fasa University of Medical Sciences in 2013 were studied. Samples were selected through census. Data collection was done using a researcher-made questionnaire using 10-point Likert scale and a checklist with Delphi technique.  Content validity of the questionnaire and its reliability were confirmed by the experts’ opinion and Cronbach's alpha of 80%.  The data were analyzed through SPSS software version 16, using both descriptive and inferential statistics (mean and standard deviation, standard score (SQ), t-test, ANOVAs). A significance level of <0.05 was considered.

**Results:**

The highest educational priority was in the field of mental health (SQ= 0.38), and environmental and professional health was the lowest priority (SQ= _0.24). Additionally, within each of the areas above specific priorities were determined. Based on the results of this study, gender, graduation date, cooperation time, and university they were educated in did not affect expressing educational needs (p>0.05). The most educational conformity with continuing education was in the diseases area (topic 27%, content 37%). In the areas of environmental and professional health and health education, compliance was zero.

**Conclusions:**

The physicians stated that mental health was the first educational need and environmental and professional health was the last one. According to the results, proper continuing medical programs should be coordinated with educational needs

## Introduction

Health is the core of all human societies and is of special importance in developing different pillars of the society. The health care system includes all organizations, institutions and people offering health services (1). The family physician project is amongst the successful programs that can eliminate the weaknesses of the health care system. The general physician and his team are responsible for ensuring people’s health and following with their treatment. One of the most important duties of family physicians is to represent joint health-remedial services. Furthermore, a family physician provides services for the population under coverage with health services actively (2). Health service package lies in level 1 in family physician’s commitments, including prevention, health promotion, treatment, referral, and management (3). On the other hand, it is obvious that a health care system is successful if its trainees are capable of responding to the needs of the society (4). Continuous learning and updating information and skills in health issues dealing with health of the society is of double importance (5), i.e. this issue is applied by continuous education. Various considerations have indicated that physicians who are far from educational environments lack enough confidence in their clinical diagnoses (6). This is a big challenge to professionals and experts and requires precise planning.

Continuous educational planning and its development should be based on the learners’ needs which are associated with physicians’ professional activity (7). Needs assessment is an important instrument in designing, developing and evaluating education programs. In the process of education, educational requirement is defined as the difference between present performance and optimal performance, the distance between what is now and what must be (8-11).

Educational needs assessment is defined as identifying educational needs and grading them according to the priority and the selection of the needs which should be reduced and/or eliminated (12). Identifying needs at various levels can lead to the increase and improvement of the quality of medical and health education and, and as a result, to greater effectiveness and efficiency of health systems, which is a part of the government’s policy to develop continuing medical education (1, 13).

Analyses done by the continuing education office of medical society have indicated that needs assessment of continuing education has been conducted partially and mainly based on the experts’ opinions and the most typical dissatisfaction of the medical society has been related to lack of coordination between job requirements and physicians’ clinical problem, and issues stated in programs (13). This study aimed to determine the family physicians’ educational needs regarding health and its applicability in continuous medical education in Fasa University of Medical Sciences.

## Methods

This was a cross-sectional study that was conducted in two stages. In the first step, we determine the educational needs of family physicians and in the second stage, educational proprieties of physicians were compared with continuing medical education programs. In order to collect sufficient data on the subject matter, it was decided to consider all employed physicians in the family physician project in Fasa University of Medical Sciences. Therefore, there was no need to determine the sample size. All participants were informed about the research and had not previously participated in similar studies. There weren’t any restrictions on the choice of subjects matched for age and sex. The study population consisted of 45 general physicians working at family physician program in Fasa University of Medical Sciences. In this study, a researcher-made questionnaire and a checklist were the data collection instruments which included demographic questions and educational subjects and they were rated from 1 to 10 levels in six different health areas. These tools were compared with continuing medical, educational programs in the recent two years. The content validity of the questionnaire was assessed by experts in the field. In order to assess the reliability of the questionnaire, repeated measure, test and Cronbach's alpha coefficient (80%) were used. In the preparation checklist, educational priorities were compiled as educational subjects were extracted according to the experts’ opinion. These educational subjects were listed by applying agreement-assessment approaches by using the Delphi technique. 

Areas considered in determining these priorities and subjects were based on the instructions, duties, expectations and responsibilities and the performance of family physicians. Based on reviews done on similar studies and texts and using physicians’ instructions, the subjects’ bank was completed. Subjects on various health areas were determined as follows:

-Health education (7 subjects)
-Mental health (6 subjects)
-Epidemic and non-epidemic diseases (10 subjects)
-Environmental and professional health (8 subjects) 
-Network management and development (10 subjects)
-Family and population health (14 subjects)

 The obtained checklist was classified for educational subjects in terms of physicians’ needs, starting from the smallest to the largest points in 10-point scales. Data analysis was obtained through descriptive statistics, mean and standard deviation, To obtain more accurate results, scores were standardized by the following formula standard score(SQ), t-test, ANOVAs for evaluating the relations. A significance level of <0.05 was considered. 

## Results

In this research, 45 general practitioners, including 20 (44.4%) men and 25 (55.6%) women, participated. The mean age of the participants was 38.3 years. The first specific purpose of this research was to determine the priority of needs education in health areas.  The results showed that mental health is the highest priority and environmental and professional health is the lowest priority (Table 1).

**Table 1:** Educational needs of family physicians on health areas in order of priority

Health areas	Mean±SD	SQ	Priority
Mental health	6.40±2.34	0.38	1
Family health	5.7±2.03	0.098	2
Diseases	5.6±1.6	0.06	3
Health education	5.2±2.4	_0.125	4
Network management and development	5.03±2.2	_0.21	5
Environmental and professional health	5.0±2.01	_0.24	6

 The second aim of this study was to evaluate educational priorities in each area (Table2). 

**Table 2:** Educational priorities in specific area

Areas	Educational subject	Mean±SD	SQ	Priority
Environmental and professional health	Health violations	6.1±3.0	0.2	1
	Ergonomy	5.6±3.1	0.032	2
	Cholorinometery	5.4±2.6	_0.038	3
	Harmful factors in workplace	5.0±2.8	_0.17	4
	Health filing of farmers, husbandries and carpet-and routine examinations	4.9±2.9	_0.20	5
	Water sanity and diseases transferred via water	4.7±2.8	_0.28	6
	Unions routine tests and approving unions & worker health card	4.2±2.7	_0.48	7
	Foods health	4.1±2.7	_0.51	8
Family health areas	Revival of children and adults	6.6±2.9	0.37	1
	Modern ways of pregnancy prevention	6.4±3.4	0.26	2
	Physical activities guide for middle-aged people during health and disease period	6.2±2.8	0.25	3
	Elderly treatment and overall elderly care	6.1±2.9	0.20	4
	Death care system program for children 1 to 59 months and executive instructions	6.1±2.9	0.20	4
	Grouping children death reasons based on ICD 10	6.1±2.9	0.20	4
	Pregnant mothers care and special care	6.0±3.4	0.14	5
	Children nutrition from birth to 8 years and supplement drugs	5.9±3.1	0.12	6
	Pap smear experiment and its interpretation	5.8±3.6	0.083	7
	Before pregnancy care	5.3±2.9	_0.068	8
	Healthy child cares	5.3±3.4	_0.058	8
	Promotion of breast feed plan and indications of powdered milk prescription	5.2±3.1	_0.096	9
	Interpretation of routine experiments before marriage	4.8±2.4	_0.291	10
	Evaluating child in risk signs	4.5±2.8	_0.357	11
Health education	Education evaluation	5.6±3.0	0.033	1
	Education methods	5.4±2.9	_0.034	2
	Educational planning	5.4±3.1	_0.032	2
	Educational media and technology	5.3±2.6	_0.076	3
	Determining problems related to health	5.1±2.9	_0.137	4
	Educational need assessment and determining priorities	4.8±2.8	_0.25	5
Diseases education	Prevention & remedy of diseases common in human and animal (Enosis)	6.1 ±2.7	0.22	1
	Thalassemia and strategies 1, 2, 3 related to it.	5.9±2.3	0.17	2
	Diseases epidemiologic examination and conducting the research	5.8±2.7	0.11	3
	Diseases transferred via sexual intercourse and it's prevention ways	5.8±2.7	0.11	3
	Diseases epidemiologic examination and conducting the research	5.8±2.7	0.11	3
	Aids and its instructions (instruction, following and remedy)	5.7±2.6	0.076	4
	Following and curing of epidemic diseases such as diabetes	5.6±2.7	0.037	5
	Tuberculosis & DOTS strategy	5.5±2.6	0	6
	Following and curing of epidemic diseases such as cancers	5.4±2.5	_0.04	7
	Following and curing, non-epidemic diseases such as hypertension	5.2±2.5	_0.12	8
Mental health	Identification, diagnosis and treatment of mental diseases	6.6±2.8	0.39	1
	Addiction prevention	6.6±2.8	0.39	1
	Instruction on mental health integration in PHC (Primary Health Care)	6.4±2.9	0.31	2
	Suicide prevention	6.3±3.0	0.26	3
	Life skills and child-growing	6.1±3.0	0.2	4
Network development and management	Instructions and criteria of family practitioners plan	5.8±3.0	0.1	1
	The way to attract people’s contributions and involving them in planning	5.7±3.1	0.064	2
	Family file and health file	5.3±3.1	_0.064	3
	Vital astronomical tables	5.2±3.0	_0.1	4
	Health system general policies	5.1±3.0	_0.13	5
	Forms and books common in health units	4.9±2.6	_0.23	6
	Health priorities	4.8±3.1	_0.22	7
	City health center duties	4.8±2.6	_0.26	7
	Taking reception from hospital	4.5±2.6	_0.38	8
	Reference system	4.2±2.7	_0.48	9

In conclusion, the above table shows that in environment and professional health (Article 13, health violations 6.6±2.9), in family health (Revival of children and adults, 6.6±2.9), in health education (Education evaluation, 5.6±3.0), in diseases education (Prevention and remedy of diseases common in human and animal, 6.1±2.7), in mental health (identification, diagnosis and treatment of mental diseases and addiction prevention, 6.6±2.8), in network development and management (instructions and criteria of family practitioners plan, 5.8±3.0) have the highest priority for family physicians.

The third aim was comparison of the existing continuous medical education programs with desired programs of family physicians. The most educational conformity with continuing education was in the diseases area. In the areas of environmental and professional health and health education, compliance was zero (Table 3).

**Table 3:** Percentage of compliance of the content and titles of existing continuing education programs with the desired one

Health Areas	Percentage of compliance	
	Content	Topic
Family health	20	13
Diseases	37	20
Environmental and professional health	0	0
Network management and development	2.5	12
Health education	0	0
Mental health	5	16

The other goal of the research was to determine the relationship between demographic data and educational needs. The results of this study showed that there was no significant correlation between sex and their educational needs. However, the t-test indicated a significant correlation between sex and their needs in” Determining problems related to health (p=0.012)” and “Educational need assessment and determining priorities” (p=0.015) in Health education area. The correlation between age and educational needs was evaluated. The result of t-test indicated a significant correlation between age and their needs in” Pregnant mothers’ care and special care (p=0.038) “in family health area and “Tuberculosis and DOTS strategy” (p=0.042) and “Aids and its instructions” (p=0.043), in disease education area. Overall, there was no significant correlation between sex and other family physicians’ educational needs. Also, the t-test indicated no significant correlation between cooperation as the family physician and graduation time from university with their educational needs.  The ANOVA test indicated no significant correlation between the types of university with their educational needs. Overall, the results of this study showed no significant relationship between gender, age, graduation time from university, and cooperation as the family physician with their educational needs. 

## Discussion

This research aimed to determine the educational needs of family physicians in Fasa University of Medical Sciences. It was found that physicians generally felt more need to mental health concepts than other areas. This can indicate that problems such as addiction, suicide, mental diseases, mental supports, and life skills are among the problems that physicians face more within our society. Results similar to these findings were observed in the study by Shiri et al. (3). Family physicians have expressed their need mostly in the mental health area. In a research conducted in Australia entitled “determination of general physicians’ educational needs”, psychiatry has been declared among the first two priorities of general practitioners’ educational need (14).

The first priority in this area lies in identification, diagnosis and treatment of mental health and addiction, in that due to the importance of mental health and its integration with network system and the issue of screening and referencing, it is felt that such issues should be taken into account with regards to education of practitioners. In a study on education of executive instructional topics in medical, educational courses, the most education was on health priorities and health system general policies and the last was on article 13 health violations and insurance criteria (15).

In this study, similar results were obtained which indicated that educational subjects of article 13 health violations is the first priority in the area of environmental and professional health. Other studies showed that educational programs have been consistent at 21% with diagnostic needs, 16% with remedial needs, and 25% with health needs (6, 8).

The results of this research stated that in the area of family health, revival of children and adults was a necessity in education for physicians which must be taken into account in the future planning. In the area of health education, the first priority is related to the education assessment. At present, according to the duties and responsibilities of family physicians, health education, whether individual or in groups, should follow scientific principles so as to have a useful effect on the society in which familiarity with proper principles of education for physicians is essential.

In the area of diseases, what the physicians stated was that prevention and treatment of diseases common among human and domestic animals (zoonosis) has the first priority. Nevertheless, the aforementioned needs can be area-oriented and such a needs assessment can be effective in the quality of continuing education in each area. A considerable problem in this area is the importance of regional needs assessment in each area. As Thomson et al. indicated, educational needs are different in various parts of the USA. There is evidence that practitioners’ re-training needs are different in urban and rural regions, as demonstrated in the study of Curran et al. (16).

Physicians in the area of management considered the educational subjects of instructions and circular letters of the family physician project as their first priority, showing that family practitioners must be more familiar with principles, conditions, duties and instructions of family physicians. 

## Conclusion

It can be concluded that physicians who work as the family physician need education in various areas of their performance, which is not included in their educational curriculum and after graduation; trainings are not consistent with special needs and national guidelines.

The present study had positive points such as participation of family physicians, agents and authorities and founders of the family physician project that guarantees its generalizability in the city and can pave the way for further research, but due to the broadness of educational subjects it was limited to special areas that influence the inclusion of all physicians’ educational needs and opinions. Thus, based on the above evidence one of the reasons for low effect of re-training can be related to the lack of an efficient needs assessment pattern in programs that should be revised and conducted by educational and official institutes with the cooperation of educational groups. 

## Notes

**Financial Disclosure: **None declared.
